# Flexible viewing time when estimating time-to-contact in 3D parabolic trajectories

**DOI:** 10.1167/jov.21.4.9

**Published:** 2021-04-26

**Authors:** Borja Aguado, Joan López-Moliner

**Affiliations:** 1 Vision and Control of Action (VISCA) Group, Department of Cognition, Development and Psychology of Education, Institut de Neurociències, Universitat de Barcelona, Barcelona, Catalonia, Spain

**Keywords:** 3D perception, calibration, internal models, optic flow, prior knowledge, TTC

## Abstract

Obtaining reliable estimates of the time-to-contact (TTC) in a three-dimensional (3D) parabolic trajectory is still an open issue. A direct analysis of the optic flow cannot make accurate predictions for gravitationally accelerated objects. Alternatively, resorting to prior knowledge of gravity and size can provide accurate estimates of TTC in parabolic head-on trajectories, but its generalization depends on the specific geometry of the trajectory and particular moments. The aim of this work is to explore the preferred viewing windows to estimate TTC and how the available visual information affects these estimations. We designed a task in which participants, wearing an head-mounted display (HMD), had to time the moment a ball in a parabolic path returned at eye level. We used five trajectories for which accurate temporal predictions were available at different points of flight time. Our results show that our observers can predict both the trajectory of the ball and TTC based on the available visual information and previous experience with the task. However, the times at which our observers chose to gather the visual evidence did not match those in which visual information provided accurate TTC. Instead, they looked at the ball at relatively fixed temporal windows depending on the trajectory but not of TTC.

## Introduction

The time remaining before an object reaches a point of interest is called time to contact (TTC). To estimate this parameter accurately is of great importance, as it can be used for multiple actions such as avoiding collisions, intercepting moving targets, and, more generally, regulating one's own speed.

Previous literature has focused on the study of TTC for objects under different visual conditions such as objects moving at a constant speed in the frontolateral plane (Bootsma & Oudejans, 1993; Tresilian, 1994) or moving toward the observer (Heuer, 1993; Lee, 1976; Wann, 1996). In contrast, during the past decades, others have focused on the study of gravitationally accelerated objects in free-fall (Lacquaniti & Maioli, 1989; McIntyre et al., 2003; McIntyre, Zago, Berthoz, & Lacquaniti, 2001; Zago et al., 2004), parabolic motion in head-on trajectories (de la Malla & López-Moliner, 2015), or frontoparallel ones (Jörges, Hagenfeld, & López-Moliner, 2018; Jörges & López-Moliner, 2019). However, the estimation of TTC for objects describing parabolas in the more general case has not been systematically addressed. This is probably due to the complex mapping between the distal three-dimensional (3D) trajectory and the projected optic variables. The same optic pattern can be caused by a multitude of sources in the external world (Pizlo, 2001), rendering the interpretation of the real source of stimulation an ill-posed problem known as the inverse problem of vision (Kersten, Mamassian, & Yuille, 2004). This problem would compromise an agent's performance based on optic information alone.

A paradigmatic case in which the computation of TTC for a 3D flying object is key is the so-called *outfielder problem*. In baseball, players known as *outfielders* must catch a flying ball at a specific time and location, avoiding ground contact. The distances involved in this task and the size of the ball render binocular cues and retinal expansion nondiscriminable. Therefore, it is usually assumed that only a reduced set of monocular cues is available to guide action (Cutting & Vishton, 1995; Wilson, Golonka, & Barrett, 2013; Zago, McIntyre, Senot, & Lacquaniti, 2008). To control the ball, some people advocate to keep task-relevant sources of visual information invariant (Chapman, 1968; Fink, Foo, & Warren, 2009; McBeath, Shaffer, & Kaiser, 1995). For example, Chapman (1968) realized that all an observer had to do to control the ball is to keep the elevation angle (γ), that is, the vertical angle between ball's position and an observer's eye level, at an increasing rate without passing over an observer's head. These strategies override the need to estimate parameters of the task such as the interception area or TTC (Shaffer & McBeath, 2005). Nevertheless, this solution is strongly dependent on concurrent sensory feedback. Because of that, it fails to explain catching behavior when the observer must turn the gaze from the ball to run directly toward the interception area or look around for a partner (Belousov, Neumann, Rothkopf, & Peters, 2016).

It is known that continuous visibility is not particularly necessary to make a catch (Chodosh, Lifson, & Tabin, 1995). Instead, all that seems necessary to catch a ball at hand is to see a portion of the trajectory large enough to integrate positional and motion estimates (Elliott, Zuberec, & Milgram, 1994) before the last 200 ms in order to avoid sensorimotor delays (Keele & Posner, 1968; López-Moliner, Brenner, Louw, & Smeets, 2010; López-Moliner & Keil, 2012; Sharp & Whiting, 1974). In this line, an alternative question arises: Is there any moment when viewing the ball is most beneficial?

Previous mathematical analysis of the information provided in the optic flow suggested that there are certain privileged positions that an observer can exploit to judge the remaining TTC. For example, in the case of juggling or catching a ball in a parabolic trajectory, kinematic information around the apex would provide privileged information to predict the remaining TTC (Todd, 1981; Watson, Banks, von Hofsten, & Royden, 1992; Whiting, 1968). This hypothesis was tested showing that, actually, the observers do not actively search for a *particular position* during the course of the parabola. Instead, they use fixed visibility windows to time the interceptive action when online visual information is not available or reliable (Amazeen, Amazeen, Post, & Beek, 1999; López-Moliner & Brenner, 2016; López-Moliner, Brenner, Louw, & Smeets, 2010). However, a problem with the studies reported above is that the balls were always thrown toward the observer and the flight time was relatively short (e.g., less than a second). Therefore, actual predictions could be overridden by learnt mappings between visual information and the remaining TTC without the need to invoke computations by an internal model (Zhao & Warren, 2015). In fact, the mathematical analysis mentioned above was carried out in terms of Cartesian variables that, unlike optic variables, are not readily available to human experience. Therefore, it is first necessary to explain how to move from optic to Cartesian variables in order to estimate the remaining time to contact.

Over the past years, a growing body of literature has advocated for the combined use of optic and prior information for the estimation of TTC. A priori knowledge of an object's size (Hosking & Crassini, 2010; López-Moliner, Field, & Wann, 2007; López-Moliner & Keil, 2012) and gravitational acceleration (Brouwer, Lopez-Moliner, Brenner, & Smeets, 2006; McIntyre, Zago, Berthoz, & Lacquaniti, 2001; Saxberg, 1987) has been suggested to calibrate the optic space into actionable estimates of TTC (Gómez & López-Moliner, 2013) and approaching speed (López-Moliner, Field, & Wann, 2007). Along these lines, Gómez and López-Moliner (2013) proposed the so-called GS model. The GS model resorts to a combination of contextual variables (ball size s and gravitational acceleration g) that are assumed to be constant and known by the observer along with time-dependent monocular cues present in the optic flow. The GS model resorts to retinal size (θ), the elevation angle (γ), and its rate of change ($\dot{\gamma }$) to obtain accurate estimates of TTC for head-on trajectories.

(1) $$\begin{array}{c} {\text{TTC}}_{\text{GS}}\approx \frac{2}{g}\frac{s}{\theta }\frac{\dot{\gamma }}{cos(\gamma )}\; \end{array}$$

**Figure 1.**
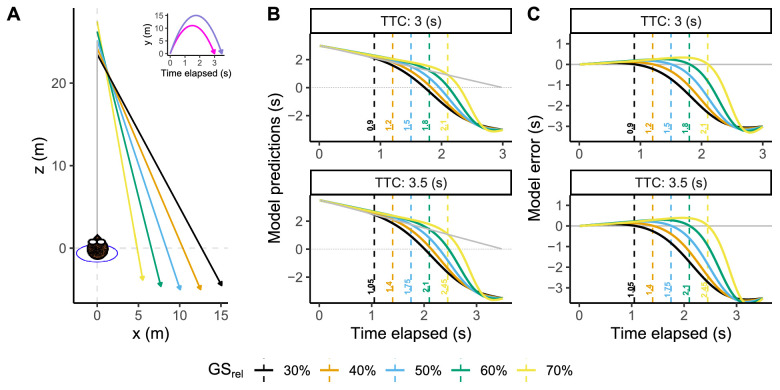
(A) Top-view representation of the five different trajectories tested in the study and a head-on approach (gray line). (B) Predictions of remaining TTC for each trajectory as a function of time elapsed since motion onset using the GS model. Gray line constitutes near-perfect accuracy for a head-on trajectory using the GS model. (C) Prediction error for each trajectory using the GS model. Positive errors indicate an overestimation of the remaining TTC and vice versa.

A priori known ball size (s) combined with retinal size (θ) maps retinal variables into Cartesian metrics. On its part, gravitational acceleration (g) normalizes the rate of change of the elevation angle ($\dot{\gamma }$) into meaningful estimates of TTC under arbitrary gravity values. As a result, the GS model is an algorithm capable of signaling accurately TTC for trajectories on a head-on approach throughout the entire flight (Gómez & López-Moliner, 2013). This is the case for the trajectory represented by a gray (thiner) line in Figure 1.

However, when a projectile is not landing on the observer, the GS model predictions are no longer accurate during all of the ball flight, because the elevation angle (γ) does not increase during the whole trajectory. As an example, in Figure 1A, the reader can see a top-view representation of five different parabolic trajectories. Each trajectory presents a different lateral offset and two different flight durations (see inset in Figure 1A). Figures 1B,C represents respective predictions and predicted errors of the remaining TTC across time using the GS model. There the reader can see that the predictions drawn by the GS model depend on the geometry of the corresponding trajectories ($GS_{\text{rel}}$) and flight duration (TTC). When the ball is launched at eye level, the GS model signals the remaining flight time accurately. Afterward, the remaining flight time is overestimated (positive errors). At a certain time for each trajectory, the predictions become accurate for a short period of time (indicated in Figure 1B,C with dashed lines). After that, the model severely underestimates the remaining flight time, rendering the predictions invalid.

Figure 1B shows that these model predictions could be used as a starting point to analyze if people can exploit the information used by the model to estimate TTC, which can be performed accurately at different proportions of flight time (see the key legend in Figure 1). As an example, the visual information contained in the model would allow to estimate the remaining TTC accurately by 1.5 or 1.75 s after launch for the blue trajectory depending on the duration of the flight. These privileged time points correspond with a 50% of total flight time passed (as indicated by $GS_{\text{rel}}$ = 50%). In principle, if an observer is sensitive to the information included in the GS model and is able to improve the performance based on feedback by using such privileged windows, it seems reasonable to assume that the observer will look actively at the ball during these temporal windows.

In this study, we will explore whether observers are able to predict TTC for parabolic trajectories of 3 and 3.5 s restricting the ball's visibility to an initial window of 300 ms and a second midflight window of 400 ms that depends on the observer's actions in each trial. With this manipulation, we will explore the preferred windows for the estimation of the remaining flight time. In that regard, the GS model can be used as a reference since it provides a different privileged temporal point to estimate the remaining flight time for each trajectory. Then, we will explore if the observers can estimate the remaining flight time with the visual information available when they look at the ball. This will allow us to test if their predictions conform to the GS model.

## Methods

### Participants

In this experiment, we tested 12 participants (*n* = 12; 4 self-identified women). They were between 21 and 32 years old. They had normal or corrected-to-normal vision. All of them were naive to experimental goals and volunteered to take part in the experiment. This study is part of an ongoing research program that has been approved by the local ethics committee of the University of Barcelona in accordance with the Code of Ethics of the World Medical Association (Declaration of Helsinki).

### Materials

#### Apparatus

**Figure 2.**
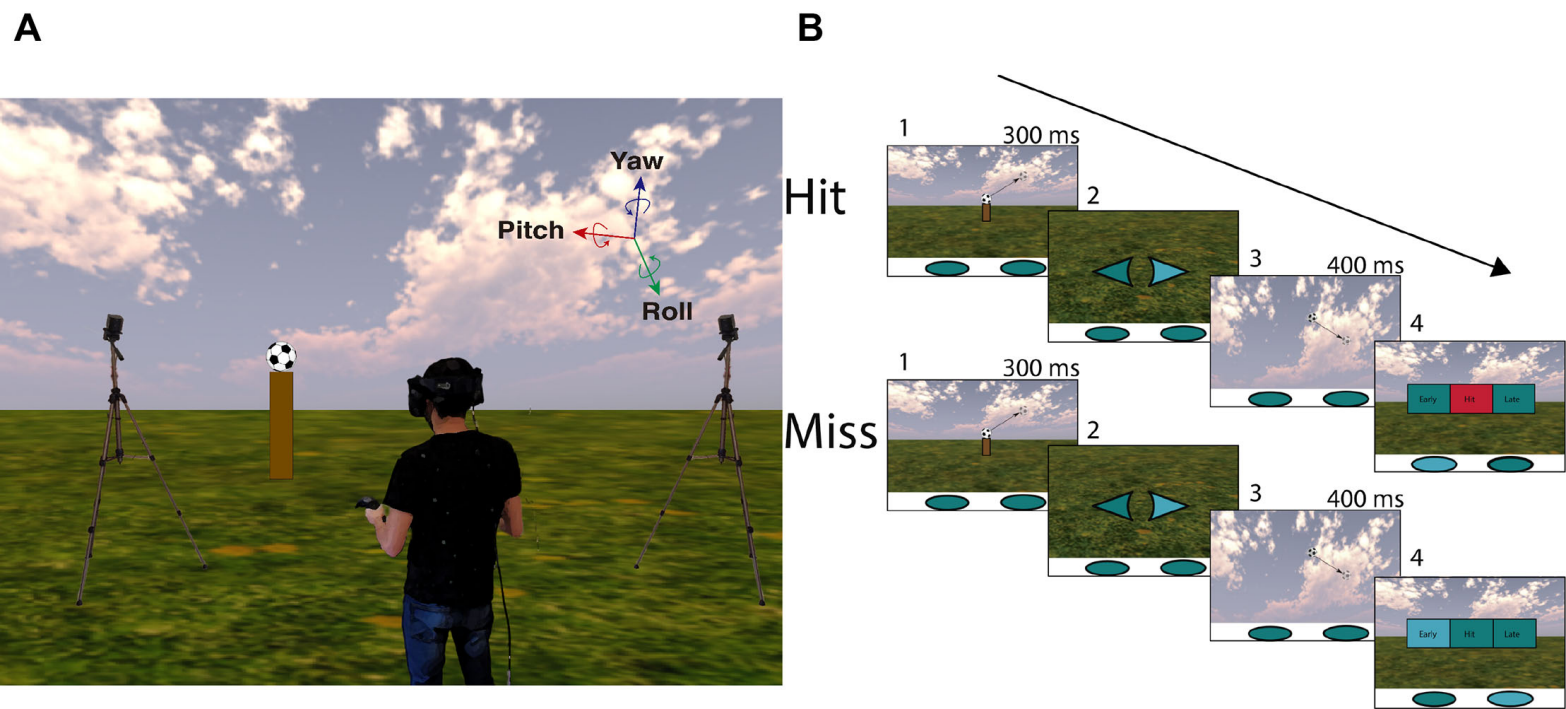
(A) Representation of the back view inside the environment of the experimental setup. (B) Representation of the course of two typical trials. 1: The ball is visible for 300 ms after launch. 2: When looking at the floor, an arrow lights up indicating the correct controller for use in the timing task. 3: The viewer decides when to look up again for the ball (visible for 400 ms). 4: The observer receives feedback about the temporal task (“early,” “good,” or “late”) and the use of the correct controller (blue or red panels).

The experiment was run by an Intel i7–based PC (Intel, Santa Clara, CA). The stimuli were rendered by an NVIDIA GeForce GTX 1070 and sent for display to an HTC Vive Pro head-mounted display at 90 Hz per eye. HTC Vive Pro has a field of view (FOV) of about 100 horizontal degrees and 110 vertical degrees. Head position and rotation were recorded by two SteamVR BASE STATION 2.0 at 90 Hz (see a representation of the setup in Figure 2A).

**Figure 3.**
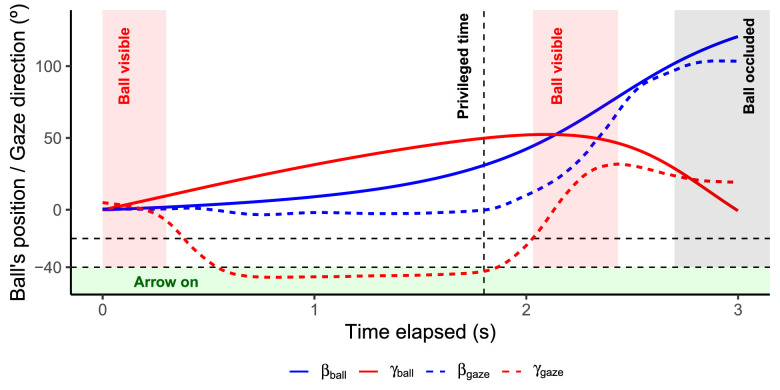
(A) Representation of the ball's position (solid lines) and gaze direction (dashed lines) across time. Blue and red lines indicate horizontal and vertical angles, respectively. The red area indicates the ball's visibility windows. Note that the midflight visibility window depends on an observer's gaze (threshold at −20 degrees). The green area indicates the visibility of the arrows on the floor (threshold at −40 degrees). The ball was always occluded 300 ms before returning at eye level again (horizon).

Head rotation was defined using a three-axis system: yaw, pitch, and roll. Assuming that the observer keeps the roll axis constant, the rotation over the pitch axis corresponds to a change in gaze vertical direction, and rotation over the yaw axis corresponds to horizontal changes in gaze direction. Since we will be referring to the ball's angular position and gaze direction, hereafter we will refer to yaw as $\beta_{\text{gaze}}$ (horizontal) and pitch as $\gamma_{\text{gaze}}$ (vertical) to ease the interpretation of results (see Figure 3). Note that gaze is inferred from the position of the head; no eye-tracking device was used.

Latency between head movement and visual feedback is about 30 ms, whereas controller latency is about 15 ms (Chénéchal & Goldman, 2018). Both should render almost negligible effects in the case of the present experiment because we are using flight times of 3 and 3.5 s.

#### Stimulus

Our stimulus consisted of a soccer ball (radius = 0.11 m) moving frontally along the three dimensions on parabolic trajectories. The ball was frontally and vertically aligned with the observer's eye level at the beginning of each trial to account for posture changes during the experiment.

Flying time was set to 3 s for five out of six trials. However, in order to prevent observers from developing rhythmic responses, a second flight time of 3.5 s was randomly interleaved in a proportion of one out of six trials.

We explored the 10 different trajectories shown in Figure 1A corresponding with a combination of five different initial positions in depth (${\text{Z}}_{\text{init}}$ = [23.49, 24.23, 25.16, 26.24, 27.47] m) and two flight times (see inset within Figure 1A). Each initial distance corresponds with a lateral final position relative to the observer (${\text{X}}_{\text{end}}$ = [15.09, 12.53, 10.06, 7.71, 5.53] m either *left* or *right*) and depth position (${\text{Z}}_{\text{end}}$ = [−4.53, −5.01, −5.03, −4.63, −3.87] m) both with a final height at eye level. Each trajectory was tailored to predict perfect accuracy by the GS model at different temporal moments corresponding to a [30, 40, 50, 60, 70] % of the flight time elapsed, that is, [0.9, 1.2, 1.5, 1.8, 2.1] s and [1.05, 1.4, 1.75, 2.1, 2.45] s for trajectories of 3 and 3.5 s of flight time, respectively.

**Figure 4.**
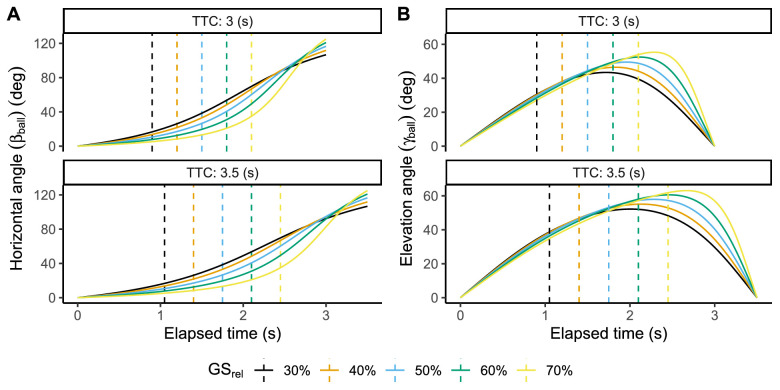
Horizontal and vertical ball's angular position for the trajectories present in this study under both flight durations. Dashed vertical lines indicate the privileged time points specified by the GS model.

A detailed scene was stereoscopically displayed providing cues for relative distance, retinal size (θ), and cardinal motion angles (horizontal: $\beta_{\text{ball}}$; vertical: $\gamma_{\text{ball}}$). Note that from now on, we will use $\beta_{\text{ball}}$ and $\gamma_{\text{ball}}$ to denote the ball's angular position in the horizontal and vertical axes with respect to the observer and ball's initial position. See Figure 3 for a combined representation of the ball's angular position and gaze direction. Figure 4 represents $\beta_{\text{ball}}$ and $\gamma_{\text{ball}}$ across time in our experimental trajectories. Note that horizontal angles larger than 90 degrees indicate that the ball is behind the observer under the initial frame of reference, assuming that the observer does not rotate. Gravitational acceleration was set at 1 *g* ($9.807\;\text{m}/{\text{s}}^{2}$, the standard at sea level). Complex dynamic effects such as air resistance and Magnus effects were neglected. Therefore, horizontal and depth movement remained constant during the same trial. No embedded rotation was simulated.

### Procedure

The main events present in the task can be described as follows (for a graphic representation see Figures 2B and 3):

(1) A soccer ball appeared frontally aligned with the observer. After 450 to 550 ms standing still on a pole, the ball was launched and remained visible for 300 ms (first visibility period in Figure 3). Note that the ball kept moving in space even when nonvisible.
(2) After this 300-ms interval, the participants were instructed to look at the floor, where there was an arrow lighting up to indicate the proper commander in that trial to perform a temporal judgment. The arrows remained visible if the head's angle was less than −40 degrees in $\gamma_{\text{gaze}}$ ($\gamma_{\text{gaze}}$ within the green zone in Figure 3).
(3) While looking at the floor, they were instructed to freely decide when to look for the flying ball. As soon as they looked up (crossed a fixed threshold at −20 degrees of $\gamma_{\text{gaze}}$), the ball reappeared and remained visible for a fixed period of 400 ms (second visibility period in Figure 3).
(4) The ball was always occluded 300 ms prior to returning at eye level (horizon). After the ball's occlusion, the observers had to estimate when the ball would return at eye level by pressing a button with the proper commander. A trial was considered a hit if the error of the temporal judgment was within ± 50 ms (3.3% of 3 s). We provided feedback for both the use of the proper commander and whether the response corresponded to a hit or a miss (early or late response).

All subjects completed six blocks of 120 trials in length. Each trajectory was presented 24 times per block (20 test trials and 4 control trials in which flight time was 3.5 s). The participants completed between 15 and 30 training trials before the main experimental procedure in order to familiarize themselves with the task. The researcher did not act as a model for the participant, that is, he did not solve the task in the presence of the participant. Instead, the participants were always encouraged to “*explore different strategies*” and encouraged to “*freely choose when to look up for the ball to obtain relevant information for the timing task*.”

### Data preparation and analysis

Data preparation and analysis were performed using R (R Core Team, 2020). We filtered out outliers using a 1.5 Interquartile Range (IQR) interval based on the response time grouped by subject, trajectory, and flight duration. This procedure removed 342 trials (3.96% of the total).

We checked that the ball's relative direction with respect to the observer did not influence the preferred viewing time (*t*(8,295.7) = −0.403, *p* = 0.686) or response time (*t*(8,263.8) = −0.459, *p* = 0.646), the two main variables in our study. Then, for the sake of simplicity, we used the absolute value of all the spatial related quantities for further analysis and visual representations.

Then, we tested our hypothesis using linear mixed modeling functions from the *lme4* (Bates, Mächler, Bolker & Walker, 2015) package. We used linear mixed models to discriminate between effects in the whole population (*random effects*) and effects for each experimental condition or subgroup in the population (*fixed effects*). Furthermore, we used a Deming regression present in the *mcr* (Manuilova, Schuetzenmeister, & Model, 2014) R package to fit linear models with measure errors in both x- and y-axes.

## Results

### Could the observers predict the trajectory?

In our experiment, the ball reappeared in the second period of visibility for just 400 ms. Therefore, the observers should predict the future ball's position, picking up information within the first 300 ms.

Due to our experimental design, the ball reappeared when vertical gaze direction ($\gamma_{\text{gaze}}$) crossed a fixed threshold of −20 degrees. Thus, we cannot use this variable directly to check the quality of the prediction. Alternatively, if our participants were able to predict the ball's vertical position, the vertical rate of change in gaze (${\dot{\gamma }}_{\text{gaze}}$) at the ball's reappearance would be linearly related to the position of the ball in the vertical axis ($\gamma_{\text{ball}}$). To test this hypothesis, we adjusted a linear mixed model with the ball's vertical angular position ($\gamma_{\text{ball}}$) as a predictor (fixed effect) of the vertical rate of change in gaze (${\dot{\gamma }}_{\text{gaze}}$).

Furthermore, if our observers were able to predict the ball's position in the horizontal axis, the horizontal position of the ball ($\beta_{\text{ball}}$) at reappearance must be linearly related to the horizontal direction of gaze ($\beta_{\text{gaze}}$). To test this hypothesis, we adjusted a linear mixed model in which we used the ball's horizontal angular position ($\beta_{\text{ball}}$) as a predictor (fixed effect) of horizontal gaze direction ($\beta_{\text{gaze}}$).

For both models, we introduced participant as a random effect. The linear mixed models were specified as

(2) $$\beta_{\text{gaze}}∼\beta_{\text{ball}}+\left(1|\text{Participant}\right)$$

(3) $${\dot{\gamma }}_{\text{gaze}}∼\gamma_{\text{ball}}+\left(1|\text{Participant}\right)$$

To check whether our participants used their predictions to guide their gaze, we compared the previous test models with null models only including the random term:

(4) $$\beta_{\text{gaze}}∼\left(1|\text{Participant}\right)$$

(5) $${\dot{\gamma }}_{\text{gaze}}∼\left(1|\text{Participant}\right)$$

**Figure 5.**
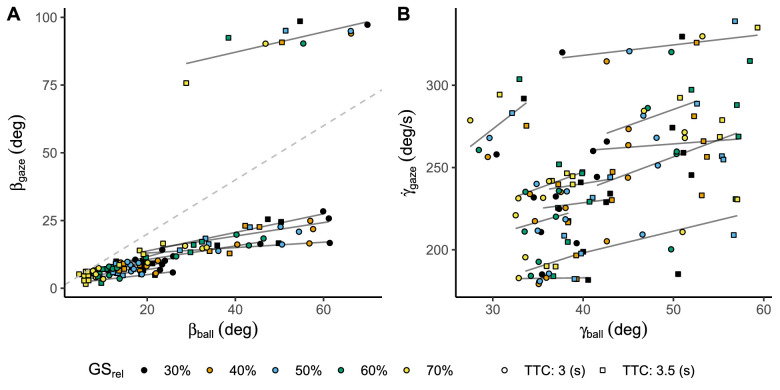
(A) Gaze horizontal position as a function of the ball's position at reappearance. (B) Gaze rate of change in the vertical axis as a function of the ball's position in the vertical axis. Both panels depict angular measures. Each trajectory ($GS_{\text{rel}}$) is indicated with a different color, whereas shape indicates flight duration (TTC). The gray lines denote the best linear fit per participant. The gray dashed line in Panel A represents the identity line.

A likelihood ratio test showed that both test models are significantly better at predicting gaze direction for the horizontal meridian ($\chi^{2}$(1) = 1,460.82, *p* < 0.001, $BIC_{\text{Null}}$= 62,443, $BIC_{\text{Test}}$= 60,991) and the vertical meridian ($\chi^{2}$(1) = 12.70, *p* < 0.001, $BIC_{\text{Null}}$ = 89,759, $BIC_{\text{Test}}$ = 89,756), indicating that our participants were able to predict different ball positions across trajectories. This pattern can be interpreted in Figure 5, in which horizontal gaze direction ($\beta_{\text{gaze}}$) and vertical rate of change in gaze direction (${\dot{\gamma }}_{\text{gaze}}$) are linearly related to the position of the ball in both axes ($\beta_{\text{ball}}$ and $\gamma_{\text{ball}}$).

**Figure 6.**
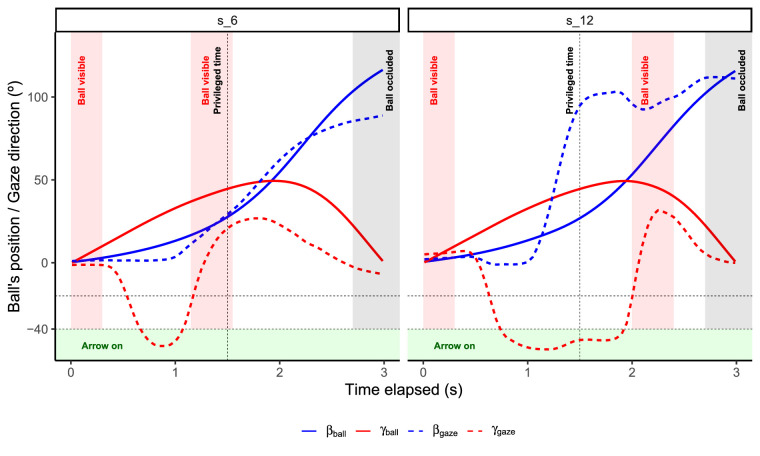
Representation of the ball's position (solid lines) and gaze direction (dashed lines) in the horizontal (blue) and vertical (red) axes across time for two different participants, **s_6** and **s_12**.

A closer look at Figure 5A points out that one of the participants (**s_12**) seems to behave differently from the rest. To illustrate in which sense the behavior of **s_12** is different from that of the average subject, we produced Figure 6. Figure 6 represents how the ball's position and gaze direction unfold across time for the average participant (**s_6**) and **s_12**. The average participant (**s_6**) keeps the horizontal gaze direction ($\beta_{\text{gaze}}$) fixed until the participant decides to look for the ball. From that moment on, the participant tries to keep the ball centred horizontally for the rest of the trial. In contrast, **s_12** rotates horizontally close to the position where the ball will fall at eye level while the ball is still occluded. Then, prior to the ball's reappearance, **s_12** predicts the ball's current position and tries to keep the ball centered horizontally during the visibility window.

### When do participants prefer to look at the ball?

Do different trajectories influence the moment at which the observers prefer to look for the ball? To analyze if this were the case, we tested whether our participants would change their preferred viewing time (from now on $t_{\text{Visible}}$) across flight duration (TTC) and trajectory ($GS_{\text{rel}}$). The preferred viewing time is defined as the first point in time at which the ball reappears from motion onset (t=0). To do this, we fitted a linear mixed model predicting the preferred viewing time ($t_{\text{Visible}}$) with trajectory ($GS_{\text{rel}}$) and flight duration (TTC) plus their interaction as predictors (fixed effects). Participant was introduced as a random effect.

(6) $$t_{\text{Visible}}∼TTC*GS_{\text{rel}}+\left(1|\text{Participant}\right)$$

To test whether our participants exploited different windows depending on the trajectory, we compared the test model with a null model only including the random term:

(7) $$t_{\text{Visible}}∼\left(1|\text{Participant}\right)$$

**Figure 7.**
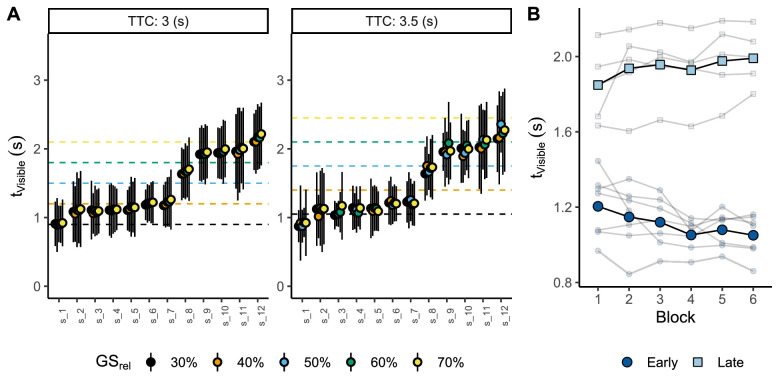
(A) Mean preferred viewing time ($t_{\text{Visible}}$) per flight duration (TTC in different panels), participant (x-axes), and trajectory ($GS_{\text{rel}}$ in different colors). The horizontal dashed lines indicate the privileged time points specified by the GS model. The error bars denote ± standard deviation. (B) Mean preferred viewing time ($t_{\text{Visible}}$) across blocks per participant (small dots) and group of participants (big dots).

A likelihood ratio test proved that the test model fits significantly better than the null model ($\chi^{2}$(3) = 65.68, *p* < 0.001, $BIC_{\text{Null}}$ = −2,763, $BIC_{\text{Test}}$ = −2,802). These results indicate that our participants decided when to look for the ball depending on the conditions present at launch (see Figure 7A).

Our results point out that the trajectory influenced when the participants preferred to look for the ball ($GS_{\text{rel}}$ : coefficient = 0.118, SE = 0.017, t = 6.872, 95% CI [0.085, 0.151]). However, neither flight duration (TTC: coefficient = 0.029, SE = 0.022, t = 1.308, 95% CI [−0.014, 0.072]) nor the interaction term ($TTC\times GS_{\text{rel}}$: coefficient = −0.016, SE = 0.042, t = −0.383, 95% CI [−0.098, 0.066]) reached significance. Hence, our results indicate that our participants adjusted the preferred viewing time to the trajectory but not to the duration of the flight, which is not unexpected since one value of flight duration (3 s) was more frequent than the other (3.5 s) on a ratio of five trials against one.

As the reader can recall, the GS model allows for accurate estimates of the remaining TTC for the trajectories present in this experiment at different points in time, specifically, at time points from 30% to 70% (by steps of 10%) of elapsed flight duration. If our participants were exploiting these privileged time points (dashed lines in Figure 7A), viewing time should change for both: trajectory ($GS_{\text{rel}}$) and flight duration (TTC). Concretely, viewing time, $t_{\text{Visible}}$, would be centered on the corresponding (same-color) dashed lines in Figure 7A. However, by simple eye inspection of Figure 7A, one can notice that the observers use relatively fixed viewing windows and quite independent from those predicted by the GS model. This was so, despite the fact that their variability could have allowed them to discover at least two privileged time points per flight time (in Figure 7A, the error bars indicate ± a standard deviation).

It could be argued that our observers did not adjust the ball's visibility to different flight durations because there was not enough information to predict TTC in the initial period of visibility. At motion onset, the rate of change of the elevation angle (${\dot{\gamma }}_{\text{ball}}$) is larger for longer flight durations, which translates into higher trajectories. Therefore, at the ball's reappearance, the rate of change in vertical gaze direction (${\dot{\gamma }}_{gaze}$) should be different between flight durations, and this was exactly the case (t(2,010.6) = −4.612, *p* < 0.001). Therefore, our observers predicted differences in the ball's vertical position across different flight durations. This result points out that the information needed to estimate different flight durations was available from motion onset.

Further exploratory analysis showed that our participants could be divided into two different groups. Those who looked at the ball before 1.5 s since motion onset (*early viewers*; from **s_1** to **s_7**) and those who looked after 1.5 s (*late viewers*; from **s_8** to **s_12**). For the sake of simplicity, participants’ labels were ordered according to the average time at which the ball reappeared, with **s_1** preferring to see the ball earlier and **s_12** preferring to see the ball later on average. In this regard, a visual comparison across blocks seems to indicate that the participants changed the viewing time throughout the experiment, emphasizing the differences between the two groups (see Figure 7B). These results are consistent with previous studies in which the observer is free to choose when to gather visual information. Under these conditions, an observer usually explores different viewing windows but ends up exploiting a fixed visibility time window to solve the task (López-Moliner & Brenner, 2016).

### Is response time affected by different flight times?

We have just shown that participants can discriminate both flight durations with the initial information at the ball's launch. However, our participants used a relatively fixed viewing time to see the ball. In the same line, we should test whether their temporal responses could be explained by using some constant criterion, mapping their actions with a fixed time to solve the task. This simple strategy, however, would result in the same response time for both flight times. Instead, if they were using some prediction based on the last moments of visibility, we would expect different response times for the two flight durations. To test this hypothesis, we fitted a linear mixed model predicting ${\text{Response}}_{\text{Time}}$ with flight time (TTC) as predictor (fixed effect) and participant as a random effect.

(8) $${\text{Response}}_{\text{Time}}∼TTC+\left(1|\text{Participant}\right)$$

Then, we compared the previous model with a null model only including the random term:

(9) $${\text{Response}}_{\text{Time}}∼\left(1|\text{Participant}\right)$$

**Figure 8.**
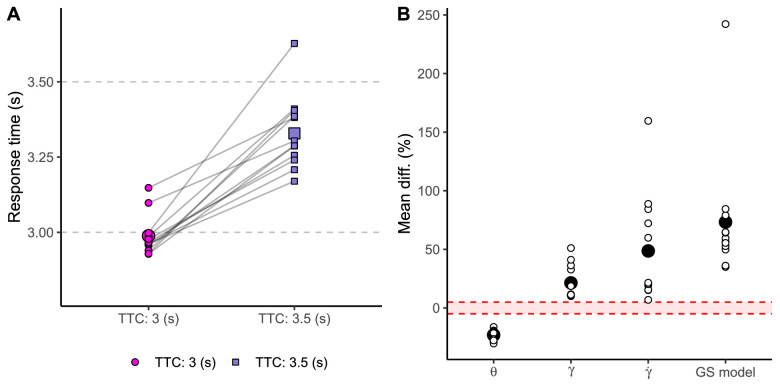
(A) Response time as a function of flight duration (color and shape code). Big dots indicate the average across participants. Smaller dots indicate the average per participant. (B) Mean difference in percentage between flight durations (TTC) per visual cue and the combination of cues (GS model). The red area denotes differences that cannot be discriminable (lower than ± 5%). Big black dots represent the average difference across participants. Small dots represent the average difference per participant.

The test model proved to fit significantly better to our experimental results than the null model ($\chi^{2}$(1) = 1,922.96, *p* < 0.001, $BIC_{\text{Null}}$ = 2,402, $BIC_{\text{Test}}$ = 488). The intercept for the test model is 2.988 (SE = 0.018, t = 162.69, 95% CI [2.954, 3.023]), and the coefficient for TTC, that is, the difference between mean response time for trajectories of 3 and 3.5 s is 0.34 (SE = 0.007, t = 46.52, 95% CI [0.326, 0.354]). Figure 8A depicts mean response time per participant and flight time (different shapes). These results show that our participants were able to use different predictive information for the two trajectories. But which sources of visual information were available to perform this prediction?

To answer the previous question, we computed the average difference for each optic cue between both flight durations in the last frame the ball was visible. Then, we obtained the proportion of change per optic cue and inferred whether an observer could use such differences to discriminate both flight durations (TTC). Previous studies have shown that human sensitivity to changes in retinal size (θ), elevation angle (γ), and the rate of change of the elevation angle ($\dot{\gamma }$) is very high, discriminating differences of ∼5% (Weber fraction) (McKee, 1981; McKee & Welch, 1992; Regan & Kaushal, 1994). Thus, we use this value as a threshold to infer if each visual cue might be readily available to be used for prediction. Our results, as depicted in Figure 8B, indicate that all the optic cues considered were discriminable between flight durations. In this sense, since all the variables included in the GS model can be discriminated, our results sustain the possibility of combining optic variables in the form of the GS model, which, as well, results in a discriminable output for all participants. But how well did early and late viewers estimate the remaining flight time?

**Figure 9.**
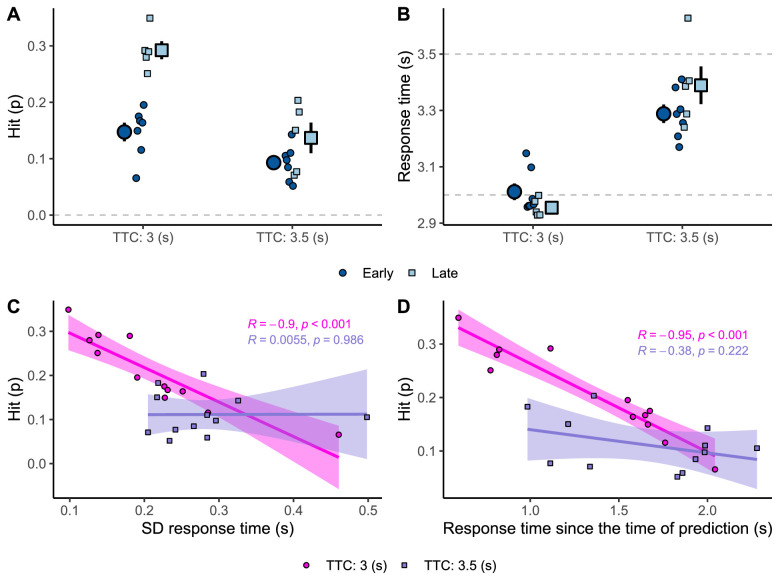
(A) Hit probability per flight duration (TTC) and group of participants. (B) Response time per flight duration (TTC) and group of participants. In Panels A and B, the big dots indicate average per group of participants, and the smaller dots indicate the average per participant. (C) Hit probability as a function of the *SD* of the response time per participant and flight duration (TTC). (D) Hit probability as a function of the response time since the time of prediction per participant and flight duration (TTC).

For both flight durations, late viewers reflect a better performance in terms of hit rate (Figure 9A). However early viewers are more accurate estimating 3-s trajectories (Figure 9B). This seems rather contradictory. Thus, what could explain this pattern? Besides the mean error, we also need to take into account the precision in the response (error variability). Figure 9C shows hit probability against the standard deviation (*SD*) of response time. The most precise participants were also the most successful for trajectories of 3 s. This can be explained by the decay of prediction on the response variability (de la Malla & López-Moliner, 2015). To confirm this possibility, Figure 9D displays the hit probability as a function of the response time since the time of prediction. We defined the time of prediction as the midpoint within the visibility window at which the observer preferred to look at the ball. For 3-s trajectories, those who relied on longer predictions also presented the lower hit probability. Therefore, the prediction decay may explain why late viewers presented a higher hit probability.

For 3.5-s trajectories, most participants underestimated the remaining TTC (Figure 9B). This underestimation can be explained by the mean response time being pulled down toward the more frequent TTC. In this case, the early viewers seemed to underestimate more the remaining TTC than late viewers, explaining their performance in terms of hit probability for trajectories of 3.5 s (Figure 9A).

### Are their estimates consistent with the use of information in the GS model?

**Figure 10.**
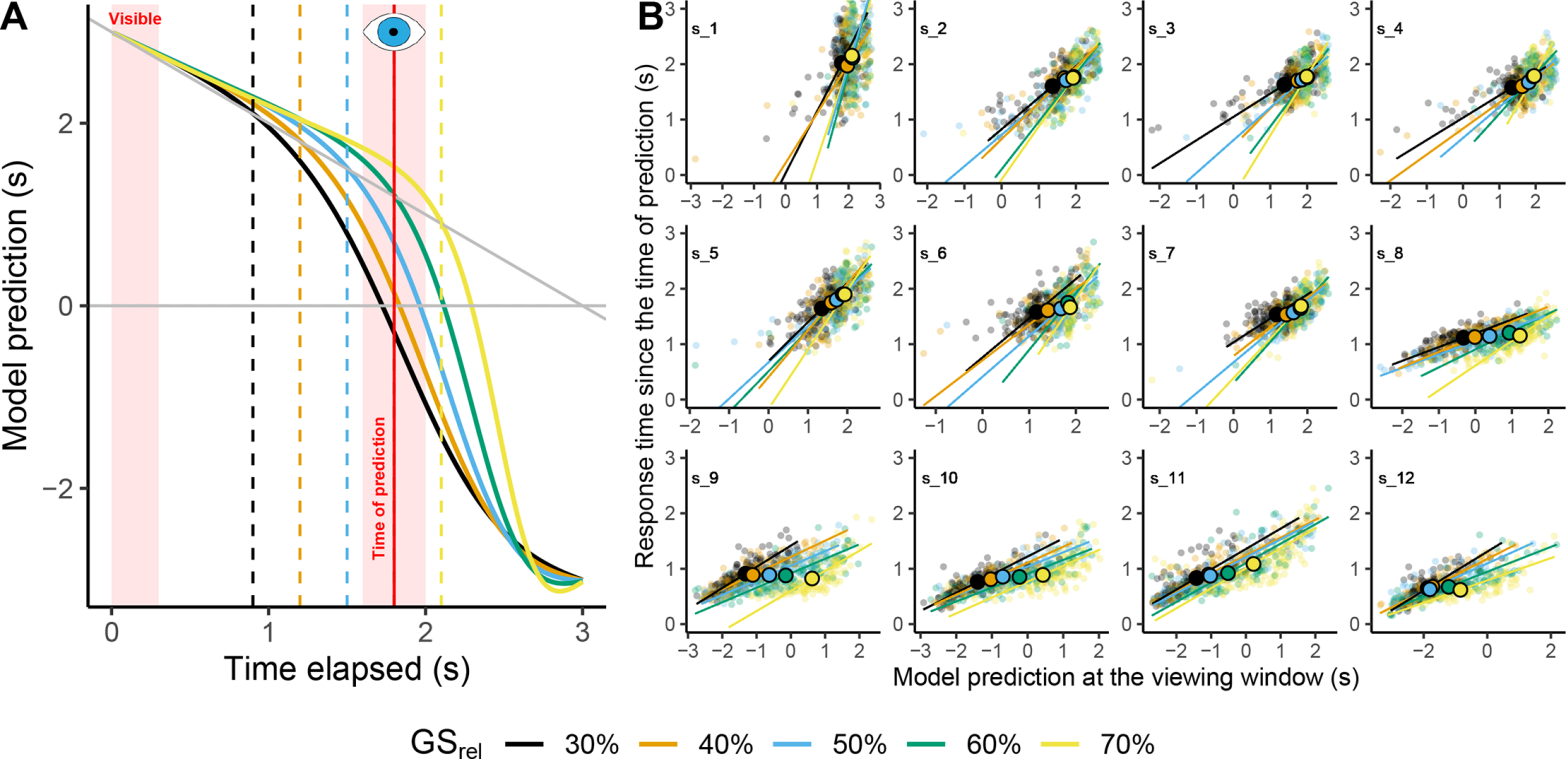
(A) Predictions of remaining flight time using the GS model as a function of the time elapsed since motion onset and trajectory. The dashed vertical lines indicate the privileged time points specified by the GS model. The vertical red line indicates an example of midpoint visibility time. (B) Response time since the time of prediction as a function of the predictions of the model at the viewing window per trajectory (color code) and participant. The colored lines indicate single fits per trajectory. Big dots indicate the average per participant and trajectory.

Our participants seem to be using relatively fixed temporal windows to look for the ball instead of the privileged viewing times specified by the GS model. Besides that, the predictive capacities of the model allow us to study the correspondence with our participants’ responses. Figure 10A reminds us of the model predictions: The predicted remaining flight time as a function of the time elapsed since motion onset. In the example in Figure 10A, the vertical red line denotes the midpoint of the visibility window (∼200 ms after ball's reappearance). We computed this moment on a trial-to-trial basis as the time at which the observer gather visual information to draw predictions about the remaining flight time.

To test if the estimated remaining time at the viewing window correlates with the prediction of the model, we fitted a Deming linear model to the response time since the central moment of the viewing window as a function of the prediction of the model at the same viewing window. We did so per participant and trajectory ($GS_{\text{rel}}$). Individual fits are represented in Figure 10B. The confidence interval for the pooled slopes across participants and trajectories does not contain zero (95% CI [0.387, 0.852]). This result points out that initially there is a relation between the response time since the viewing window and the predictions of the GS model.

Furthermore, the remaining flight time predicted by the model is different across trajectories: The vertical red line (viewing window) intersects the prediction of the GS model at different predicted values per trajectory. Concretely, the GS model predicts longer flight durations for those trajectories in which the privileged time takes place later after motion onset (e.g., yellow line in Figure 10A). Unlike the previous analysis in which we fitted a linear model within trajectories, we now fitted a linear model across trajectories. We used a linear mixed model with the trajectory ($GS_{\text{rel}}$) as fixed effect (predictor) and participant as random effect. The fitted slope is positive and different from 0 ($GS_{\text{rel}}$: coefficient = 0.322, SE = 0.025, t = 13.111, 95% CI [0.273, 0.371]). These results can be inferred in Figure 10B, in which the average response time since the viewing window is larger for those trajectories where the privileged time takes place later. However, one participant (**s_9**) does not seem to follow this overall trend (see Figure 10B). Because of that, it is important to note here that we cannot rule out the possibility that an observer may be using simpler strategies based on mapping some available optic cues to the temporal response (Zhao & Warren, 2015).

Finally, since the GS model predicts different moments for which the estimation of the remaining flight time is accurate, we decided to check whether people looking at the trajectory closer to those privileged windows leads to some benefit in terms of the temporal error. To do so, we used the median to split the trials per participant and trajectory depending on how close/far they looked at the trajectory with respect to the privileged time specified by the GS model (i.e., 50% closer vs. 50% far). The mean error for the closer trials (−0.031 s) is smaller than the mean error for the far trials (−0.045 s) (t(8,264.7) = 2.371, *p* = 0.018). Therefore, these results indicate some benefit for when the observer looks at the trajectory closer to the privileged time points specified by the GS model.

## Discussion

We humans normally track a ball to catch it on the fly. Nevertheless, there are times when this is not possible because we need to divert our gaze somewhere else. By studying the behavior and performance mimicking these situations, we might gain important insights into how people cope with these scenarios. In this work, we analyze a task in which observers had to judge TTC on the basis of a short periods of visual information. This is a very demanding task in which the participants had to predict the ball's position in flight in order to capture the necessary information to estimate the remaining TTC. Our task tries to capture situations in which players control a ball in a parabolic flight while looking elsewhere searching for a teammate.

If possible, an observer will try to reduce the use of an offline (predictive) strategy by always keeping the ball visible (Brenner & Smeets, 2011; Mazyn, Savelsbergh, Montagne, & Lenoir, 2007). Continuously updating predictions increases reliability (Snowden & Braddick, 1991) and lessens perceptually driven errors (Belousov, Neumann, Rothkopf, & Peters, 2016; Binaee & Diaz, 2019; de la Malla, Smeets, & Brenner, 2018), which explains why our behavior changes when unexpected perturbations occur (Fink, Foo, & Warren, 2009). However, the present task requires a predictive component at all stages.

Congruently with previous literature (Diaz, Cooper, Rothkopf, & Hayhoe, 2013; Jörges & López-Moliner, 2019), our findings show evidence that people can successfully use visual information from a short visibility period at motion onset to predict future balls’ positions. After that, restricting midflight visibility to a brief window forced our participants to focus on and exploit useful visual information for the estimation of TTC. Previous studies had already shown that it is only necessary to see a short portion of the trajectory to catch a ball (López-Moliner, Brenner, Louw & Smeets, 2010; Whiting & Sharp, 1974). But here we provide further evidence that there is enough information to estimate TTC despite the viewing window that is chosen to extract visual information (see: Is response time affected by different flight times? and Are their estimates consistent with the use of information in the GS model?). In this line, the estimations of the remaining flight time are related to the predictions by the GS model and consistently biased across trajectories in the expected direction (see: Are their estimates consistent with the use of information in the GS model?). However, we cannot rule out the possibility that an observer may be using simpler strategies based on temporal mappings between different optic cues and the temporal response (Zhao & Warren, 2015).

Gathering visual information in advance can be useful for action planning when a secondary activity has to be performed. However, in our experiment, those participants relying on longer predictions (early viewers) were also more affected by the average flight time and prediction decay. In contrast, those who preferred to see the ball at the very last minute (late viewers) showed less response variability. This difference seems to be responsible for the better performance of late observers (see: Is response time affected by different flight times?). Nevertheless, despite the preferred viewing time, all our observers preferred to look at the ball in relatively fixed time windows regardless of TTC and the privileged time point specified by the GS model (see: When do participants prefer to look at the ball?). Why did they decide to look for the ball at those specific moments?

Likely, our participants may not be sensitive to the improvement in hit rate resulting from the use of the visual information within the privileged time points. If the privileged time occurs well before the response, response variability may hinder the detection of an increase in accuracy. Moreover, due to the interleaved presentation of trajectories, our experimental design may have prompted our participants to adopt a unitary strategy to exploit the available visual information rather than taking advantage of the privileged windows specified by the GS model (Amazeen, Amazeen, Post, & Beek, 1999; López-Moliner & Brenner, 2016; López-Moliner, Brenner, Louw, & Smeets, 2010). Under this rationale, our observers would be less likely to correct the preferred viewing time. Instead, they would prefer to correct their temporal estimates on a trial-to-trial basis (López-Moliner, Vullings, Madelain, & Van Beers, 2019; Shadmehr, Smith, & Krakauer, 2010).

An important consideration concerning this study is the possible perceptual distortions introduced by immersion in virtual reality (VR) wearing a head-mounted display. A number of studies have shown that the perceived space in VR is compressed, presumably due to a conflict between accommodation and disparity signals (Hoffman, Girshick, Akeley, & Banks, 2008). This could explain why TTC is consistently underestimated when the estimations are guided by a combination of binocular variables (the rate of change of disparity) and an estimate of distance (Gray & Regan, 1998; Lages, 2006; Rolin, Fooken, Spering, & Pai, 2018). Nevertheless, the geometric layout in our experiment would reduce the impact of spatial compression since distance is uninformative of TTC in parabolic trajectories with a lateral offset. In line with the GS model, we think that cardinal monocular variables (horizontal and vertical angles: β and γ) are more likely to be used in the estimation of TTC and future positions of the ball. For example, viewing the ball peripherally may bias and reduce the reliability of elevation angle estimates (γ) (Crowell & Banks, 1996), leading to systematic errors in the estimation of the remaining flight time. However, the direction in which visual eccentricity translates into systematic errors might need to be addressed in further studies in which temporal visibility and visual eccentricity can be decoupled.

Tracking a ball visually typically requires the use of both head and eye movements (Mann, Spratford, & Abernethy, 2013). However, in our study, we only have data on the head's direction. It is known that eye movements can be quite different from head tracking in interception tasks. Usually, eye movements reflect a predictive component that seems to improve visual pursuit and reflect predictions of a future position of an object at key moments (Diaz, Cooper, Rothkopf, & Hayhoe, 2013; Jörges & López-Moliner, 2019; Mann, Spratford, & Abernethy, 2013). An analysis of eye movements could have allowed us to be more precise analyzing how the quality of visual tracking improves the estimation of TTC. Furthermore, analyzing eye movements at key moments such as the ball's reappearance or the moment the ball falls at eye level would be interesting to shed light on the predictive component of visual function for the control of interception tasks.

## Conclusion

Our data indicate that the observers are able to use a predictive strategy to estimate both the position of the ball in flight and the remaining flight time using the visual information available during short visibility windows. To estimate the remaining TTC from a midflight visibility window, our observers preferred to use fixed temporal windows that might help them to interpret visual information combined with their previous experience.
